# Identification of genes directly responding to *DLK1* signaling in Callipyge sheep

**DOI:** 10.1186/s12864-018-4682-1

**Published:** 2018-04-24

**Authors:** Hui Yu, Jolena N. Waddell, Shihuan Kuang, Ross L. Tellam, Noelle E. Cockett, Christopher A. Bidwell

**Affiliations:** 1 Department of Animal Sciences, Purdue University, 270 South Russell Street, West Lafayette, IN 47907 USA; 2 Department of Molecular and Integrative Physiology, University of Michigan, 1000 Wall Street, Ann Arbor, MI 48105 USA; 3 Center for Cancer Research, Purdue University, West Lafayette, IN USA; 4 CSIRO Animal, Food and Health Sciences, St. Lucia, QLD Australia; 5 Department of Animal, Dairy and Veterinary Sciences, Utah State University, Logan, UT USA; 6 Department of Animal Science & Veterinary Technology, Tarleton State University, Stephenville, TX USA

**Keywords:** *DLK1*, *RTL1*, Skeletal muscle, Hypertrophy, Callipyge sheep, Primary effector, Secondary effector

## Abstract

**Background:**

In food animal agriculture, there is a need to identify the mechanisms that can improve the efficiency of muscle growth and protein accretion. Callipyge sheep provide excellent machinery since the up-regulation of *DLK1* and *RTL1* results in extreme postnatal muscle hypertrophy in distinct muscles. The aim of this study is to distinguish the genes that directly respond to *DLK1* and *RTL1* signaling from the genes that change as the result of muscle specific effects.

**Results:**

The quantitative PCR results indicated that *DLK1* expression was significantly increased in hypertrophied muscles but not in non-hypertrophied muscles. However, *RTL1* was up-regulated in both hypertrophied and non-hypertrophied muscles. Five genes, including *PARK7*, *DNTTIP1, SLC22A3, METTL21E* and *PDE4D,* were consistently co-expressed with *DLK1*, and therefore were possible transcriptional target genes responding to *DLK1* signaling. Treatment of myoblast and myotubes with DLK1 protein induced an average of 1.6-fold and 1.4-fold increase in *Dnttip1* and *Pde4d* expression respectively. *Myh4* expression was significantly elevated in DLK1-treated myotubes, whereas the expression of *Mettl21e* was significantly increased in the DLK1-treated myoblasts but reduced in DLK1-treated myotubes. DLK1 treatment had no impact on *Park7* expression. In addition, *Park7* and *Dnttip1* increased *Myh4* and decreased *Myh7* promoter activity, resemble to the effects of *Dlk1*. In contrast, expression of *Mettl21e* increased *Myh7* and decreased *Myh4* luciferase activity.

**Conclusion:**

The study provided additional supports that *RTL1* alone was insufficient to induce muscle hypertrophy and concluded that *DLK1* was likely the primary effector of the hypertrophy phenotype. The results also suggested that *DNTTIP1* and *PDE4D* were secondary effector genes responding to *DLK1* signaling resulting in muscle fiber switch and muscular hypertrophy in callipyge lamb.

**Electronic supplementary material:**

The online version of this article (10.1186/s12864-018-4682-1) contains supplementary material, which is available to authorized users.

## Background

Callipyge sheep are well known for their postnatal muscle hypertrophy that is prominent in loin and hind quarters [1–3]. The muscle mass in callipyge sheep is increased 35–40% and carcass fat is decreased 6–7%, while the live weights are the same relative to normal lambs [3–6]. Callipyge lambs are born with normal muscling and hypertrophy becomes detectable at 4–6 weeks of age [2, 4, 7, 8]. After 5 weeks of age, an increased proportion and larger size of fast-twitch, glycolytic muscle fibers become apparent in callipyge muscles [7, 9]. Not all the skeletal muscles in callipyge sheep develop hypertrophy, the *supraspinatus* and *infraspinatus* muscles in thoracic limbs both do not undergo hypertrophy [1, 5, 6].

The callipyge mutation is a single base change of an A (wild-type allele) to a G (callipyge allele) between *DLK1* (*Delta-Like homolog 1*) and *MEG3* (*Maternal Expressed Gene 3*) genes in the *DLK1*-*DIO3* (*Deiodinase, Iodothyronine, type III*) imprinted gene cluster [10, 11]. The callipyge mutation does not disrupt the protein coding sequence. The highly conserved 12 bp sequence including the mutation acts as a long-range control element to alter the transcription of the surrounding imprinted genes in *cis*. Specifically, the inheritance of a callipyge allele from the sire up-regulates the transcription of the paternal protein-coding genes *DLK1* and *RTL1 (Retrotransposon-like 1)*, while the inheritance of a maternal callipyge allele enhances the expression of maternal non-coding RNA including *MEG3*, *MEG8 (Maternal Expressed Gene 8)* and *RTL1AS (RTL1 antisense*) [12–16]. As the muscling phenotype is only expressed in heterozygous individuals that inherit the mutation from the sire, it has been concluded that a paternally expressed protein encoding gene(s) must be the primary effector.

*Dlk1* encodes a transmembrane protein belonging to the epidermal growth factor (EGF)-like repeat containing family. It functions as an antagonist to down-regulating Notch signaling [17, 18]. Notch signaling is involved in conserved cell fate decisions and is known to inhibit the expression of myogenic regulatory transcription factor *MyoD* and myogenic differentiation, but enhances satellite cell proliferation and self-renewal [19, 20]. Several studies indicate that *Dlk1* expression can influence postnatal muscle growth. Transgenic mice over-expressing *Dlk1* had increased muscle mass and myofiber diameter [21]. Similarly, mice with deletion of the maternal microRNA379/544 cluster displayed muscle hypertrophic phenotype with an elevation of *Dlk1* expression, suggesting the regulation of maternal imprinted microRNA on paternal *Dlk1* gene expression [22]. Muscle-specific gene ablation of *Dlk1* in the mouse resulted in reduced body weight and skeletal muscle mass due to reduction in myofiber numbers [23]. Conversely, over-expression of *Dlk1* in cell culture inhibited myoblast proliferation and enhanced differentiation [23]. *DLK1* mRNA was up-regulated in *longissimus dorsi, gluteus medius* and *semimembranosus*, the hypertrophied muscles in callipyge lambs but not up-regulated in *supraspinatus*, the non-hypertrophied muscle [13, 15, 24, 25]. The DLK1 protein was expressed at high levels in the myofibers of callipyge *longissimus dorsi* and *semimembranosus* but was not detected in normal muscles [26, 27]*.*

Similar to *DLK1*, the mRNA abundance of *RTL1* is also increased in the callipyge muscles. *Rtl1* belongs to Ty3-Gypsy retrotransposons gene family and contains gag-pol-like structure common to retroviruses [28]. This highly conserved gene has a single exon and encodes a full length 151 kDa protein in callipyge skeletal muscles [29]. In mouse, *Rtl1* is highly expressed at the late-fetal stage in both fetus and placenta and the loss or over-expression of *Rtl1* causes late fetal and / or neonatal lethality [30]. Enhanced expression of *Rtl1* in liver has been suggested to drive hepatocarcinogenesis [31]. Interestingly, transgenic mice expressing ovine *RTL1* in skeletal muscle have significant increased mass of hind legs and *quadriceps* with larger myofibers in EDL (mostly composed of glycolytic fast twitch fibers) muscle [32]. However, in callipyge sheep, the up-regulation of *RTL1* is not specific to hypertrophied muscles, a lower magnitude of induction of *RTL1* in the non-hypertrophied muscle (*supraspinatus*) was detected [13], suggesting that *RTL1* alone is not the primary inducer for increased muscle mass in callipyge sheep. These combined studies suggest that elevated *DLK1* expression is the primary cause of callipyge muscle hypertrophy and *RTL1* probably has a synergistic effect with *DLK1.*

*MEG3*, *MEG8,* and *RTL1AS* are all non-coding RNAs that are transcribed from the maternal chromosome in the same imprinted region. These genes are host to a number of microRNA and snoRNA (*MEG8*) [33]. Murine *Meg3* was proposed to possess tumor suppressor properties [34–36] and extensive studies have been conducted recently to explore the mechanisms on how *Meg3* inhibits cell growth [37–41]. Enhanced *Meg3* expression was reported in obese mice and it further aggravated glucose intolerance in these mice [42]. Furthermore, during postnatal muscle development, *Meg3* expression level was high after birth, however, it decreased rapidly afterwards in both pigs and mice [43, 44], implying its possible role in the growth of myofibers through hyperplasia instead of hypertrophy in late developmental stages [44]. In contrast to *Meg3*, there are few studies performed on *Meg8*. It is known that *Meg8* was expressed in embryonic brain and muscles [45]. Maternal nutritional status significantly influenced *MEG8* expression in fetal *semitendinosus* muscle in sheep [46, 47]. Temple syndrome patients have hypermethylated region in *MEG8* gene [48, 49]. Nevertheless, knowledge about the exact function of *MEG8* still remains unclear. *Rtl1as* contains at least four microRNA that cause RISC-mediated degradation of *Rtl1* transcripts [50, 51]. As a result, deletion of miR-127 (one of the microRNAs processed from *Rtl1as*) increased *Rtl1* expression, leading to placentomegaly and defects in the placental labyrinthine zone [52].

Several studies have been conducted to identify transcriptome changes in the muscles of callipyge animals [24, 25, 53]. Microarray analysis of gene expression in the *semimembranosus* identified 375 genes that were differentially expressed in callipyge versus normal lambs [24]. Twenty-five transcripts were further verified by quantitative PCR [24]. It has been assumed that among these 25 transcripts, there are direct targets of *DLK1* signaling that act as secondary effectors to increase protein accretion and fiber type changes (tertiary responses) that occur during hypertrophy. The current study will distinguish the genes that appear to respond to *DLK1* signaling from tertiary effects of hypertrophy (see schematic diagram, Fig. 9a), using a broader set of hypertrophied and non-hypertrophied muscles at an age when hypertrophy is developing in the callipyge lamb. Genes that are transcriptionally responsive to *DLK1* signaling would be expected to have a similar mRNA expression pattern as *DLK1* in all the muscle types examined. In contrast, the tertiary responsive genes may not stringently follow the *DLK1* expression pattern. These tertiary response genes could show greater variability across muscle types due to differences such as metabolism, myofiber types and exercise. From this initial screening of candidate genes, the effect of DLK1 as a ligand and transfection of target genes were tested using primary mouse myoblasts to confirm transcriptional activities in myogenesis. Since part of the callipyge hypertrophy phenotype includes a shift to a greater number of fast-twitch, glycolytic muscle fibers expressing *MYH4*, then changes in *MYH4* expression was chosen as an indicator of a tertiary response.

## Methods

### Sample collection

Matings between a ram that was heterozygous for the callipyge allele and a group of normal ewes were used to generate a cohort of callipyge and normal lambs. The lambs were genotyped by detection of the callipyge SNP [10, 11] and tissue samples were obtained from four callipyge and four normal lambs at 30–35 days of age following protocols approved by Purdue University Animal Care and Use Committee. Seven muscles (*longissimus dorsi*, *semimembranosus*, *semitendinosus*, *triceps brachi*, *supraspinatus*, *infraspinatus*, and heart) were weighed after dissection. A small piece of sample was preserved in RNAlater (ThermoFisher Scientific, PA, USA) and stored at − 20 °C for further RNA extraction. Muscle samples were homogenized in 4 M guanidinium thiocyanate, 25 mM sodium citrate, 50 mM EDTA, and 1% sodium-N-lauroyl-sarcosine. The CsCl ultracentrifuge method was used to isolate RNA. Briefly, the muscle homogenate was centrifuged through a CsCl cushion (5.7 M CsCl, 50 mM EDTA) and the sedimented RNA was further purified using NucleoSpin RNA II columns (Machery-Nagel, PA, USA) with Dnase I treatment [25].

### Primary myoblast isolation and culture

Primary myoblasts were isolated from hind limb skeletal muscles of mice at 3–5 weeks of age. Muscles were washed with Dulbecco’s Phosphate-Buffered Saline (DPBS), minced and digested in type I collagenase and dispase B mixture (Roche Applied Science, Indianapolis, IN USA). The digested muscle pulp was then filtered through a 100 μm filter (CellTrics^®^, Partec Inc., Swedesboro, NJ USA) to remove large muscle fiber debris and then plated on collagen-coated dishes. After 3 days, cells were collected and digested with 0.025% trypsin for 10 min with agitation. Cells were seeded in growth media (F-10 Ham’s medium supplemented with 20% fetal bovine serum, 100 units/mL of penicillin, 100 μg/mL of streptomycin, 0. 292 mg/ml of L-glutamine, and 4 ng/mL basic fibroblast growth factor) on non-coated plates for 45 min to deplete fibroblasts, as previous described [23, 54] and then transferred to collagen (Roche Applied Science)-coated dishes. Myoblasts were differentiated into myotubes after plating cells at approximately 80% confluency on Matrigel (BD Biosciences, San Jose, CA USA) coated plates and the addition of fusion media consisting of DMEM supplemented with 5% horse serum, 100 units/mL of penicillin, 100 μg/mL of streptomycin, and 0. 292 mg/ml of L-glutamine. Myoblast cultures testing the effects of DLK1 protein were plated on a bed of 1 mg/mL BD Matrigel containing 500 ng/mL of recombinant DLK1 protein [DLK1 (mouse): Fc(human), Adipogen International Inc., San Diego CA USA. Cells were induced to differentiate the next day and fused for 2 days before mRNA isolation.

### Quantitative PCR analysis

Complimentary DNA (cDNA) synthesis for measuring *RTL1* transcript abundance used gene-specific priming of 5 μg total RNA and Superscript III reverse transcriptase at 50 °C (Life Technology). The cDNA synthesis for other transcripts used random hexamer priming from 5 μg RNA and MMLV reverse transcriptase. The first strand cDNA synthesis reaction was diluted 25-fold so that there was an equivalent of 20 ng of input RNA per microliter. Quantitative PCR assays were carried out in 15 μL reaction volumes of iQ SYBR Green Supermix with cDNA equivalent to 100 ng of input RNA. All cDNA samples were assayed in duplicate. Absolute quantification was used to measure gene expression in sheep muscle RNA. The quantitative PCR primers and the plasmid standards were designed and tested according to the methods described previously [24]. Primer sequences are listed in Additional file 1. Cloned amplicons were used as standards to calculate a regression of threshold cycle on molecule copy number to determine a log value of starting abundance for each of the cDNA samples based on their threshold cycle [13]. All plasmid standards were diluted from either 1 × 10^8^ to 1 × 10^2^ or 1 × 10^7^ to 1 × 10^1^ molecules. Quantitative PCR reactions for standards were performed in triplicate. The variance analysis was performed using SAS 9.2 (SAS Institute Inc., Cary, NC, USA) software and the MIXED procedure was used to analyze the log value of gene expression. Genotype was the main effect in the model and each muscle was analyzed individually. The random effect was defined as animal nested within genotype. The least squares means, standard error and difference between least squares means were calculated for the variance analysis.

Relative quantification was used to measure gene expression in DLK-treated myoblast experiments. RNA from cultured myotubes was extracted and purified using Nucleo Spin RNA II columns (Machery-Nagel Inc., Easton, PA USA) with DNase I treatment. First strand cDNA was synthesized from RNA using random hexamer and oligo dT priming and MMLV (Life Technologies). Quantitative PCR measurements were performed using the SA Bioscience SYBR Green Supermix (QIAGEN, Valencia, CA USA) reagents on an iCycler Real-Time PCR Detection System (Bio-Rad Inc.). Each reaction was carried out in 15 μl reaction volumes of SA Bioscience SYBR Green Supermix with 5 pM of each primer and diluted first-strand cDNA. Primer sequences are listed in Additional file 1. Ribosomal protein large protein 38 (*Rplp38*) was used as the housekeeping gene control for *ΔC*_*T*_ calculation (*ΔC*_*T*_ = *C*_*T*_ of the target gene – average *C*_*T*_ of housekeeping genes). Fold expression values were calculated using 2^-ΔΔCT^ methods [55], where ΔΔC_T_ = (*ΔC*_*T*_ of the treatment sample) – (*ΔC*_*T*_ of control treatment samples) with no added DLK1 as control treatment and normalized to 1. Statistical significance was determined by Analysis of Variance (ANOVA) method using SAS9.2.

### Plasmid construction

Full length *DNTTIP1, METTL21E* and *PARK7* cDNAs were amplified by reverse transcription PCR from total sheep RNA, directionally cloned into pENTR/SD/D-TOPO vector (Life Technologies, Grand Island, NY USA) and then subcloned by recombination into the pcDNA3.2/V5-DEST expression vector (Life Technologies), according to the manufacturer’s recommendations. The mouse pDLK1-pCMV-SPORT 6.1 plasmid was commercially available (cat#: MMM1013–9201636, Thermo Fisher Scientific Inc., PA USA). To confirm the insertion of the target genes with an intact open reading frame, all plasmids were sequenced from both directions. The pGWCAT-pcDNA3.2 /V5 control plasmid was obtained from Life Technologies.

The mouse *Myh4* luciferase construct (pGL3IIB2.6) contains 2.56 kb of the promoter region of *Myh4*, and the rat *Myh7* luciferase construct (p-3542β-MHCluc) contains 3.5 kb of the promoter of *Myh7* [56, 57]. The pRL-SV40 plasmid expressing *renilla* luciferase was commercially available from Promega Corporation. Plasmids for electroporation were purified using EndoFree Plasmid Maxi Kit (QIAGEN) and quantified by Nanodrop spectrophotometry (Thermo Fisher Scientific Inc., Rockford, IL USA.

### Luciferase reporter assay

Neon ™ Transfection System (Life Technologies) was used according to the manufacturer’s recommended protocol. Myoblasts (2 × 10^5^ cells) were electroporated with 3 μg of plasmid DNA with three pulses 10 ms pulses of 1500 V. To determine if target genes could have effects on myosin gene expression, the effector experiments were performed by co-transfection of protein coding sequence of the target genes along with the myosin luciferase reporter construct. The myosin luciferase reporter construct and the transfection control (*renilla luciferase*) were kept constant first, and the two different amounts of effector cDNA were titrated. In order to keep a constant amount of the total plasmid DNA, the GW-CAT was added and also used as the null control vector. A detailed description of the plasmid combinations are given in Additional file 2. The electroporated cells were put into 96-well plates in growth media overnight and subsequently fused into myotubes for 3 days. In *Park7* luciferase experiments, the indicated concentrations of IGF1 (50, 100, and 200 ng / mL) (long®R3 IGF1, Sigma-Aldrich Co, St Louis, MO USA) were added to the fusion medium 24 h after differentiation and cultured for another 48 h. The reporter assays were performed with Dual-Luciferase Reporter Assay System (Promega, Madison, WI USA), according to the manufacturer’s recommendations. The samples were read with a Tecan Genios Pro (Tecan Group Ltd.) plate reader. Luciferase activity was adjusted for transfection efficiency by multiplying the firefly luciferase activity of a given well by the ratio of mean *renilla* luciferase activity for all wells divided by *renilla* luciferase activity of the given well to produce units of adjusted luciferase activity. The results were analyzed for the addition of target construct as the main effect by ANOVA using SAS 9.2 software. The IGF1 treatment was also considered as a main effect when analyzing *PARK7* luciferase assay.

## Results

### Phenotypic data analysis

**Fig. 1:**
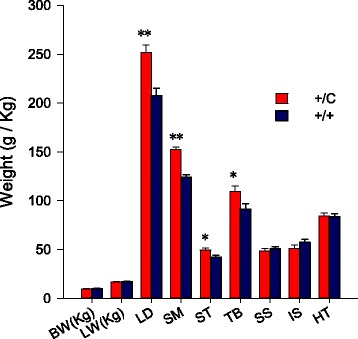
Muscle hypertrophy in callipyge sheep. There were no differences in the birth weights (BW) and live weights (LW) between callipyge (+/C) and normal lambs (+/+). Callipyge lambs had significantly heavier *longissimus dorsi* (LD), *semimembranosus* (SM), *semitendinosus* (ST) and *triceps brachii* (TB) muscles. There were no differences in muscle weights for the *supraspinatus* (SS), *infraspinatus* (IS) and heart (HT). Significant differences are indicated by (*; *P* < 0.05, or **; *P* < 0.01) between callipyge and normal lambs within each muscle

Animal birth weight and live weight were collected (Fig. 1) and statistical analysis showed that there were no significant differences in birth weight and live weight between callipyge (+/C) and normal lambs (+/+). The callipyge lambs had significantly heavier *longissimus dorsi*, *semimembranosus*, *semitendinosus* and *triceps brachi*, ranging from 23% to 17% greater relative to muscles of normal lambs (Fig. 1), thus these four muscles are referred to as “hypertrophied muscles” in this study. No significant differences were detected in *supraspinatus*, *infraspinatus*, and heart; hereafter these are referred to as “non-hypertrophied muscles”. These results verified the muscle specific phenotype of hypertrophy in the experimental samples.

### Muscle specific gene expression

The mRNA abundance of the 25 differentially expressed transcripts identified from a previous study [24] were assayed by quantitative PCR. To investigate the shift of myosin heavy chain isoforms in callipyge animals, four myosin heavy chain genes (*MYH1, MYH2*, *MYH4, MYH7*) were also examined in this study. The complete set of primers for all of the genes analyzed by quantitative PCR and the PCR cycling conditions were given in Additional file 1. The least-square means for gene expression in all seven muscles was given in Additional file 3. The *p*-values for differential expressed genes in seven muscles were listed in Additional file 4.

**Fig. 2:**
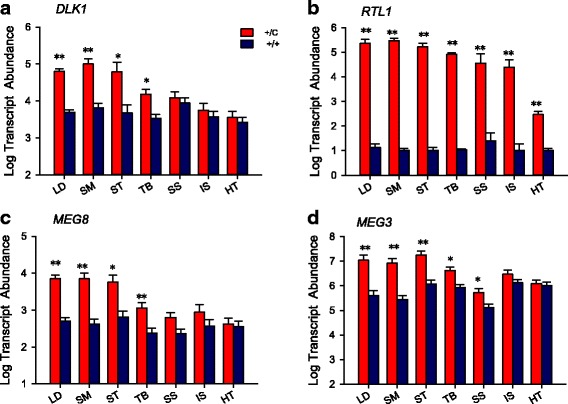
Transcript abundance of genes from the *DLK1-DIO3* locus. **a** *DLK1*; **b** *RTL1*; **c** *MEG8* and **d** *MEG3. *Least square means and standard errors for log transcript abundance are shown for each muscle and genotype, callipyge (+/C) and normal (+/+). The hypertrophied muscles are LD, SM, ST, and TB and the non-hypertrophied muscles are SS, IS and HT. The increased expression of paternally allele-specific genes *DLK1* and *RTL1*, and the maternal allele-specific non-coding RNA *MEG8* and *MEG3* in callipyge hypertrophied muscles are shown. Significant differences are indicated by (*; *P* < 0.05, or **; *P* < 0.01) between genotypes within each muscle

The expression of *Ribosomal protein, large P0 (RPLP0)* was used as a control gene. *RPLP0* expression was not significantly different in the two genotypes in all seven muscles (Additional file 5), which indicated that equivalent amounts of RNA were used for cDNA synthesis and for quantitative PCR. Compared to normal lambs, the expression of *DLK1* was significantly increased in hypertrophied muscles with *semimembranosus* having the largest magnitude of increase (11-fold); and *triceps brachi* had the smallest increase (6.6-fold). There were no significant differences in *supraspinatus*, *infraspinatus* and heart (Fig. 2a). In contrast to *DLK1*, *RTL1* expression in normal lambs was extremely low in *longissimus dorsi*, *semitendinosus*, *triceps brachi* and *supraspinatus* with an average log value of 1.14, and its expression level was barely detectable in *semimembranosus*, *infraspinatus* and heart with average log value is less than 1 (Fig. 2b). The expression of *RTL1* was significantly increased in callipyge lambs in all the assayed muscles with an average log value for 4.6. The expression patterns of *MEG3* and *MEG8* were quite similar since both of their expression levels were significantly increased in the hypertrophied muscles (Fig. 2c and d). The callipyge lambs also had significantly increased *MEG3* expression (6-fold, *P =* 0.0323) in *supraspinatus* (Fig. 2d). There was a trend towards significance in *MEG8* expression (*P =* 0.0678) (Fig. 2c). The expression patterns of these imprinted genes in the *DLK1-DIO3* locus were consistent with previous reports [13, 15].

**Fig. 3:**
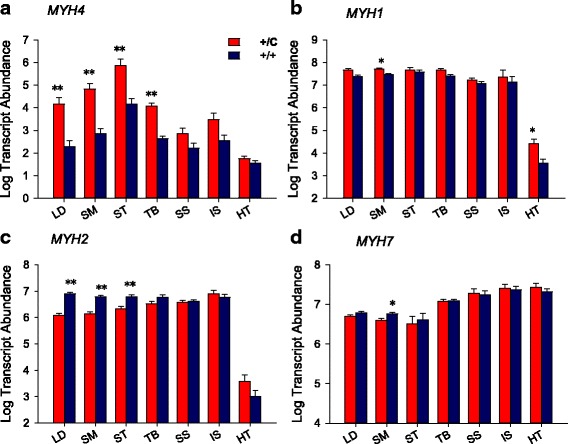
Transcript abundance of myosin heavy chain genes. **a** *MYH4*; **b** *MYH1*; **c** *MYH2* and **d** *MYH7*. Least square means and standard errors for log transcript abundance in 100 ng of total RNA are shown for each muscle and genotype. Callipyge animals indicated by +/C, and normal animals are represented by +/+. LD, SM, ST, and TB are hypertrophied muscles, SS, IS and HT are non-hypertrophied muscles. Samples are from 30 to 35 days of age lamb. Significant differences are indicated by (*; *P* < 0.05, or **; *P* < 0.01) between callipyge and normal lambs within each muscle

The expression of several myosin isoforms were examined as markers for muscle hypertrophic response. The differential expression of myosin isoforms showed the established fiber type changes in callipyge muscle. A previous study reported the significant and strong up-regulation of muscle genes characterized of type IIb (*MYH4*) and down-regulation of genes characterized of type IIa fibers (fast oxidative/glycolytic) (*MYH2*) and type 1 fibers (slow oxidative) (*MYH7*) in hypertrophied muscles [53]. The present study confirmed this phenotype by showing an average of 60-fold increase in *MYH4* expression in the hypertrophied muscles (Fig. 3a). *MYH1* is the most abundant myosin isoform measured in the skeletal muscles and its mRNA abundance was significantly increased in *semimembranosus* by 2.5-fold (Fig. 3b). *MYH2,* which characterized an intermediate fiber type between fast and slow fibers, was relatively unchanged in *triceps brachi*, but down-regulated in other hypertrophied muscles, particularly in *longissimus dorsi* (6-fold) (Fig. 3c). The mRNA abundance of *MYH7* was only down-regulated in *semimembranosus* (1.6-fold), which is different from the previous report indicating a decreased level of *MYH7* in *longissimus dorsi* (Fig. 3d) [7, 9, 53]. Overall, the myosin heavy chain gene expression results confirmed the reported callipyge phenotype with increased fast-glycolytic and decreased slow-oxidative myofibers [9, 53].

**Fig. 4:**
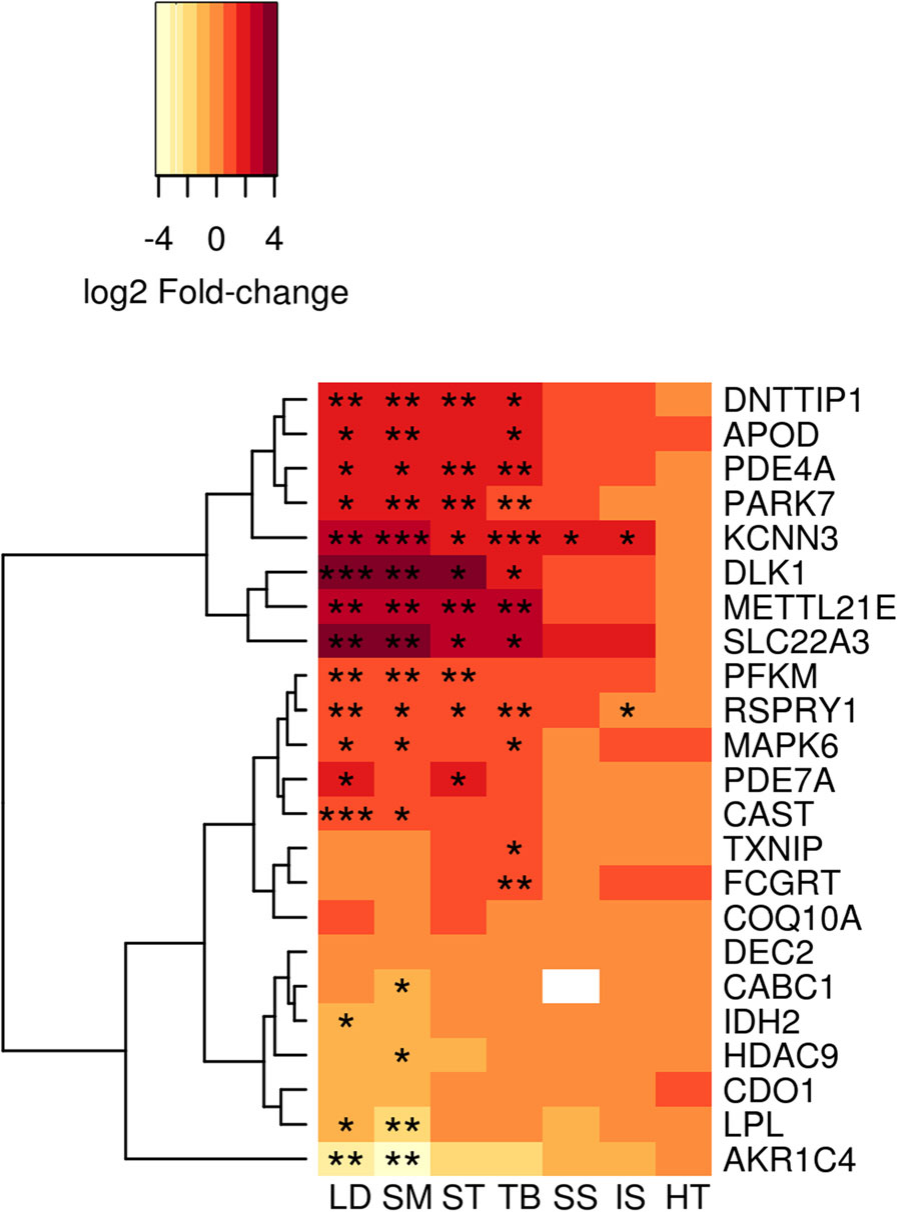
Hierarchal clustering of candidate gene expression pattern in 7 Muscles. Columns for the seven muscles were fixed and rows representing gene expression were subject to clustering. The fold change of transcript abundance for the ratio of callipyge to normal was transformed to log2. The color scale for this heat map is red-orange-yellow, where the red indicates the magnitude of the fold change in callipyge is higher than normal and yellow represents gene expression was lower in callipyge than normal muscle. Significant differences are indicated by (*P<0.05, **P<0.005 and ***P<.0005) between callipyge and normal lambs within each muscle

**Fig. 5:**
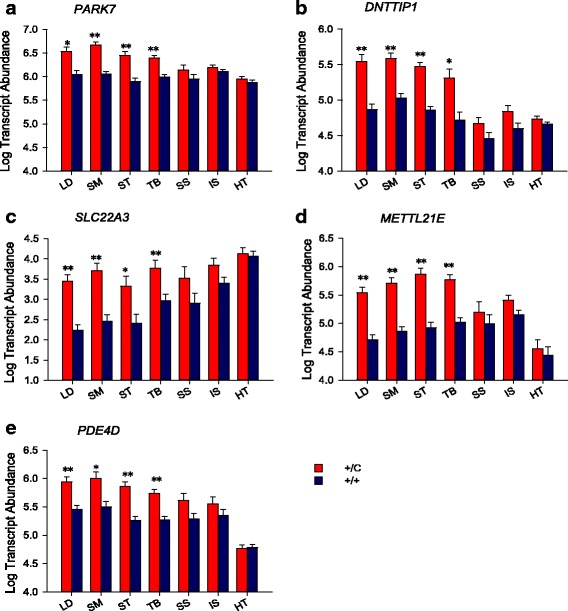
Transcript abundance of the candidate target genes in 7 Muscles. **a** *PARK7*; **b** *DNTTIP1*; **c** *SLC22A3*; **d** *METTL21E*; and **e** *PDE4D*. Least square means and standard errors for log transcript abundance are shown for each muscle and genotype, callipyge (+/C) and normal (+/+). Five genes exhibited co-expression with *DLK1* induced hypertrophy; *PARK7, DNTTIP1, SLC22A3, METTL21E*, and *PDE4D* were up-regulated in hypertrophied muscles, LD, SM, ST, and TB but not in the non-hypertrophied muscles SS, IS and HT. Significant differences are indicated by (*; *P* < 0.05, or **; *P* < 0.01) between genotypes within each muscle

Gene expression profiles for the 23 transcripts, identified from the microarray analysis [24], across the 7 muscle types are shown in Fig. 4. Five genes including *Parkinson Protein 7 (PARK7,* also known as *DJ-1)* (Fig. 5a), *Deoxynucleotidyltransferase, terminal, interacting protein 1* (*DNTTIP1)* (Fig. 5b), *Solute carrier family 22 member 3 (SLC22A3)* (Fig. 5c)*, protein-lysine methyltransferase 21E* (*METTL21E*) (Fig. 5d), and *cAMP specific phophodiesterase 4D (PDE4D)* (Fig. 5e) were specifically up-regulated in the hypertrophied muscles, but not in the non-hypertrophied muscles, resembling *DLK1* expression pattern. Therefore, these five genes are the potential target genes that may directly respond to *DLK1* signaling in hypertrophied muscles. The changes in gene expression of *PARK7* in hypertrophied muscles were small relative to the other four genes, but significantly different from normal muscles with *semimembranosus* having the biggest 6.1-fold increase (Fig. 5a). *DNTTIP1* had the biggest magnitude of increase in *longissimus dorsi* (6.8-fold) and smallest increase in *semimembranosus* (5.6-fold) (Fig. 5b). There was a substantial up-regulation of *SLC22A3* in hypertrophied muscles with the magnitude ranging from 8.1-fold to 12.5-fold (Fig. 5c). The average mRNA abundances for *METTL21E* (Fig. 5d) and *PDE4D* (Fig. 5e) in hypertrophied muscles were 8.4-fold and 5-fold greater relative to normal lambs. In addition to these five transcripts, a muscle specific expression pattern was observed for all of the genes measured (Fig. 4). In consistent with previous microarray and quantitative PCR study using *longissimus dorsi* and *semimembranosus* [24], majority of the 23 transcripts were differentially expressed in these two muscle types in the current study. Many of these genes are metabolic in nature; for example, *PFKM (phosphofructokinase, muscle)* was up-regulated in three of the four hypertrophied muscles and *LPL (Lipoprotein Lipase)* was down-regulated in two of the four hypertrophied muscles, therefore they are likely to be involved in response to the changes of *MYH* isoforms and metabolic demands in callipyge lambs rather than direct response to *DLK1* signaling.

### Effects of DLK1 on potential direct target genes and myosin heavy chain gene expression

**Fig. 6:**
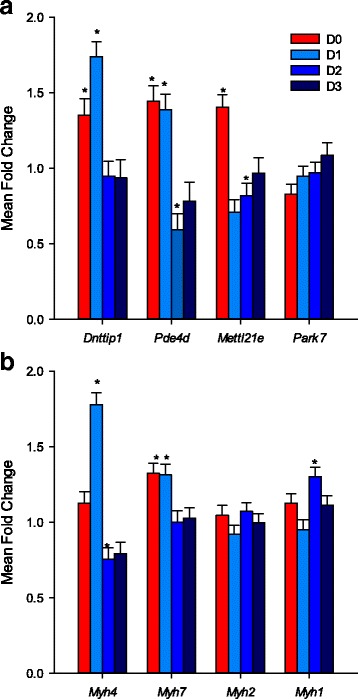
Transcript abundance of candidate genes and myosin heavy chain genes in DLK1-treated myoblasts and myotubes. Primary myoblasts were cultured on Matrigel (Control) or Matrigel plus recombinant DLK1 protein for 24 h (D0) and induced to differentiation for up to 72 h. RNA was collected at 24 h intervals; D0: myoblasts in proliferation medium, D1: 24 h; D2: 48 h and D3: 72 h in differentiation medium. **a** *Dnttip1* and *Pde4d* expression were significantly increased in DLK1 treatment at D0 and D1. *Mettl21e* expression was highly elevated at D0 but immediately decreased after differentiation (D1). The expression of *Park7* was not significantly altered by DLK1 treatment. **b** The mRNA abundance of *Myh4* was not significantly changed in myoblasts (D0) but was highly elevated after 24 h of myotube differentiation (D1) with DLK1 treatment. Expression of *Myh7* expression was significantly increased in D0 and D1. No significant differences were observed in the expression of *Myh2 and Myh1*. Significance differences (*; *P* < 0.05) between control and DLK1 treatment within differentiation time (D0-D3)

In order to examine the effect of DLK1 signaling on the expression of potential target genes, we treated myoblasts and myotubes with recombinant DLK1 protein. Since C2C12 cells over-expressing *Dlk1* failed to proliferate [23], primary myoblasts were plated onto Matrigel containing recombinant DLK1 protein to enable DLK1 to act as ligands for cell surface receptors. Primary myoblasts were induced to fuse and mRNA was collected at 24 h intervals to a maximum of 3 days (D0, D1, D2 and D3). The expression patterns of myosin heavy chain genes were also examined as controls for cell differentiation. Notably, the expression of most genes examined dramatically reduced 1 day after differentiation (D2 and D3), which may be the result of loss of DLK1 recombinant protein effects after 48 h (Fig. 6a and b). Therefore, the emphasis was put on data from myoblasts (D0) and myotubes (D1). The mean fold change of each gene for DLK1 treatment was compared to its expression level in control treatment. The mRNA abundance of *Dnttip1* was significantly increased by DLK1 treatment in both D0 myoblasts and D1 myotubes (Fig. 6a). Specifically, DLK1-treated D0 myoblasts had 1.4-fold increase in *Dnttip1* expression and the DLK1-treated D1 myotubes had 1.7-fold increase. The same trend was also observed for *Pde4d* expression with 1.4-fold increase in DLK1 treated D0 myoblasts and 1.38-fold increase in DLK1 treated D1 myotubes. The expression of *Mettl21e* has an opposite trend in myoblasts and myotubes with significant increased expression in DLK1- treated D0 myoblasts and significant reduced expression in DLK1-treated D1 myotubes (Fig. 6a). However, the mRNA abundance of *Park7* was not significantly changed in the presence of DLK1 (Fig. 6a). *Slc22a3* was tested but not reported in this assay since its expression was too low to be reliably quantified. The expression of *Myh4* was very low in D0 myoblasts and no significant differences were detected between control and DLK1 treatments (Fig. 6b). However, after differentiation, the mRNA abundance of *Myh4* was 1.8-fold (*P* < 0.0001) higher in the DLK1 treated D1 myotubes than control treatment. Surprisingly, the expression of *Myh7* was significantly increased in both DLK1-treated myoblasts and myotubes with an average 1.4-fold increase (Fig. 6b), which was opposite to callipyge lambs whose *MYH7* expression are lower than normal lambs. There were no significantly differences between DLK1 treatment and control treatment in *Myh1* and *Myh2* expression at D0 and D1.

### Effects of potential direct target genes on *Myh4* and *Myh7* luciferase activity

In order to determine whether *PARK7*, *DNTTIP1*, and *METTL21E* could influence myosin isoform expression similar to what occurs in callipyge muscle, a series of luciferase assays were conducted to test the effects of these candidate genes on *Myh4* and *Myh7* promoter activities. The luciferase assays were conducted by co-transfection of the candidate gene cDNA constructs as effector plasmids together with *Myh4* (pGL3IIB2.6) or *Myh7* (p-3542β-MHCluc) luciferase reporter plasmids in primary myoblasts. The amount of effector plasmid that was co-transfected was titrated for each plasmid with addition of null vector (pGWCAT) to maintain equal amounts of input DNA. After transfection, myoblasts were differentiated into myotubes for 3 days. The expression of effector protein from the vectors were confirmed by transfection of C2C12 cells. The cells were stained with corresponding antibodies after transfection (*DNTTIP1* in Additional file 6, *PARK7* in Additional file 7 and *METTL21E* in Additional file 8). These results validated the expression of these constructs and also illustrated the nuclear localization of DNTTIP1, and the nuclear and cytoplasmic localizations of PARK7, METTL21E and GWCAT.

**Fig. 7:**
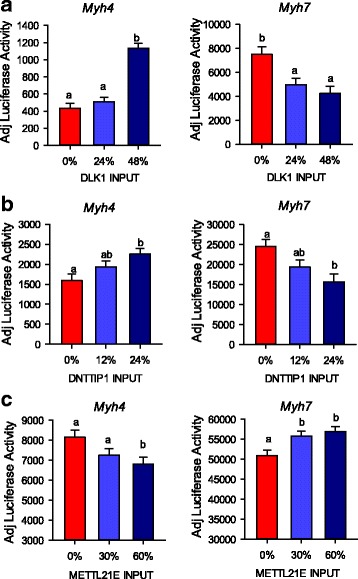
Effects of DLK1, DNTTIP1 and METTL21E on myosin promoter activity*.* Increased *Myh4* and decreased *Myh7* luciferase activity was examined in DLK1 (**a**) and DNTTIP1 (**b**) over-expressed myotubes. Over-expression of METTL21E (**c**) decreased *Myh4* and increased *Myh7* luciferase activity. Primary myoblasts were transfected with different compositions (indicated by percentage in graph) of effector constructs together with *Myh4* or *Myh7* luciferase plasmids. The pRL-SV40 plasmid was transfected into cells as a transfection efficiency control. The transfected cells were put into 96-well plates in growth media overnight and fused into myotubes for 3 days. Luciferase activity was adjusted for transfection efficiency by multiplying the firefly luciferase activity of a given well by the ratio of mean *renilla* luciferase activity for all wells divided by *renilla* luciferase activity of the given well to produce units of adjusted luciferase activity. Differing lower case letters indicate significance (*P* < 0.05) within each test

There was a dose effect for *DLK1* induced *Myh4* luciferase activity. Transfecting *DLK1* significantly increased *Myh4* luciferase activity at the high concentration (48%) (*P* = 0.0012) (Fig. 7a). The same effect was also observed in *DNTTIP1* treatment; higher concentration (24%) of *DNTTIP1* resulted in significantly increase in *Myh4* luciferase activity (*P* < 0.0001) (Fig. 7b). In this experiment, less plasmid DNA was used for *DNTTIP1* because it was a nuclear factor and predicted to have a strong effect on downstream targets. Conversely, adding *METTL21E* (60%) significantly decreased *Myh4* luciferase activity (*P* = 0.0126) by 16.5% (Fig. 7c). An opposite dose response was observed using *Myh7* luciferase assay for *DLK1* and *DNTTIP1* treatments. A 35% decrease in *Myh7* luciferase activity occurred with a high concentration of either *DLK1* (48%) (Fig. 7a) or *DNTTIP1* (24%) (Fig. 7b) treatment. Adding *METTL21E,* in contrast, significantly increased *Myh7* luciferase activity even at a low concentration (30%, *P* = 0.0135 relative to control) (Fig. 7c).

**Fig. 8:**
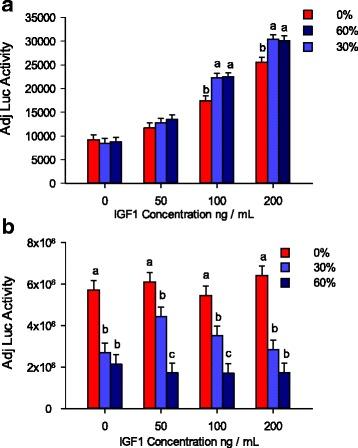
Effect of PARK7 on myosin promoter activity*.* Primary myoblasts were transfected with different compositions of effector constructs pPARK7-pcDNA3.2 and pGWCAT- pcDNA3.2 (control) plasmids together with *Myh4* or *Myh7* luciferase reporter plasmids. The pRL-SV40 plasmid served as a transfection efficiency control. Myosin promoter-luciferase reporter activity was adjusted for transfection efficiency and normalized across all samples on the plate using *renilla* luciferase activity. **a** Transfection of PARK7 effector plasmid (input 30% and 60%) significantly elevated *Myh4* luciferase activity at 100 and 200 ng/mL concentrations of IGF1; **b** Transfection of PARK7 plasmid significantly decreased *Myh7* luciferase activity regardless of IGF1 concentration. Differing lower case letters indicate significance (*P* < 0.05) within each IGF1 treatment

Since *PARK7* was assumed as a positive regulator in the PI3K/AKT pathway, which was initiated by the binding of IGF1 to its receptor [24, 58], different concentrations of IGF1 were applied to the myotubes in order to induce a differential response in *PARK7* treatment. Statistical analysis indicated the overall effect of *PARK7* was significant in both *Myh4* (*P* = 0.0004) (Fig. 8a) and *Myh7* (*P* < 0.0001) (Fig. 8b) luciferase assays. Specifically, with a higher concentration of IGF1 (100 ng/mL) treatment, adding *PARK7* even at a low concentration (30%) significantly increased *Myh4* luciferase activity (*P* = 0.0007). There is a similar trend at an IGF1 concentration of 200 ng/mL with 30% input of *PARK7* significantly increasing *Myh4* luciferase activity by 20% (Fig. 8a). No significant difference was found at low IGF1 concentration (50 ng / mL) and no added IGF1 treatment. There was a dose effect for IGF1 treatment in the *Myh4* luciferase assay, however, *Myh7* luciferase activity was unaffected by IGF1 treatment. A dose response for *PARK7* was observed in *PARK7* induced *Myh7* luciferase activity. Adding *PARK7* (60%) significantly reduced *Myh7* luciferase activity regardless of IGF1 concentrations (Fig. 8b).

## Discussion

In food animal agriculture, there is a need to identify the mechanisms that can improve the efficiency of muscle growth and protein accretion. Callipyge lambs have improved feed efficiency with greater than 35% more muscle mass, which shows that much higher muscle growth is biologically possible. Therefore callipyge muscle hypertrophy provides a unique model for investigating the genes that are potentially rate limiting for muscle growth. The transcripts examined in the current study were initially identified and validated in a previous postnatal developmental series (10, 20, 30 days and 80 days) by microarray analyses [24].

The animals in this study reproduced the established phenotype that the callipyge lambs have similar live weights as normal lambs [4, 6], and the well-recognized pattern of muscle hypertrophy with increased muscle mass in the pelvic limb and the torso relative to the thoracic limbs [3]. The gene expression analysis in the *DLK1-DIO3* locus showed the established pattern for induction of muscle hypertrophy in callipyge lambs. There were high levels of expression of *DLK1* and *RTL1* in affected muscles from the callipyge lambs. *DLK1* was only up-regulated in hypertrophied muscles but the up-regulation of *RTL1* was also detected in the three non-hypertrophied muscles. The expression levels of *MEG3* and *MEG8* were increased in *supraspinatus*. This combined evidence suggests that *RTL1* alone is insufficient to induce muscle hypertrophy and reinforces the conclusion that *DLK1* is the primary inducer of muscle hypertrophy. Transgenic mice over-expressing either *Dlk1* or ovine *RTL1* have been shown to have increased muscle mass and both genes have been shown to be targets of microRNA hosted by the maternal ncRNA genes in the *DLK1-DIO3* imprinted cluster to account for polar overdominance inheritance mechanisms [32]. Therefore, here we include *RTL1* as a possible synergistic factor to act in concert with *DLK1* to induce the callipyge phenotype.

Analysis of several myosin isoforms showed up-regulation of fast twitch glycolytic myosin isoform (*MYH4*) in all hypertrophied muscles and down-regulation of the fast twitch mixed oxidative and glycolytic myosin isoform (*MYH2*) in hypertrophied muscles except *triceps brachi*. The changes in muscle fiber types are indicative for muscle metabolism, which have been reported to be directly associated with elevated postnatal expression of DLK1 protein in muscle fibers [26]. A decrease in slow twitch oxidative *MYH7* was only detected in the *semimembranosus* but not in other hypertrophied muscles, which is inconsistent with previous observation of the decreases in *MYH7* expression and smaller oxidative myofibers in *longissimus dorsi* [7, 26, 53]. The discrepancy is likely due to the younger animals used in the current study, since the decreased level of *MYH7* is not evident at 30–35 days of age.

**Fig. 9:**
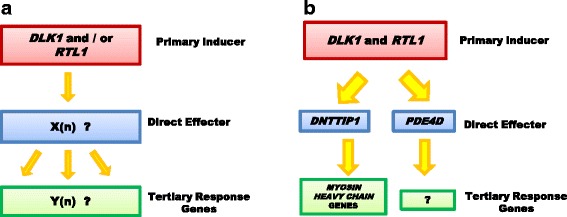
Schematic diagram showing the hypothesis (**a**) and conclusions (**b**) of this study

Among the 23 examined transcripts, five genes, *DNTTIP1, PARK7, PDE4D, SLC22A3*, and *METTL21E*, were up-regulated specifically in hypertrophied muscles, resembling *DLK1* expression pattern in seven muscles and thus these genes were considered as the secondary targets in response to *DLK1* signaling. Only *Dnttip1* and *Pde4d* were up-regulated in DLK1-treated myoblasts and myotubes suggesting a direct signaling effect of DLK1 on the transcriptional expression of these two genes. Taken together, these combined results indicated that *DNTTIP1* and *PDE4D* are potential secondary effector genes responding to *DLK1* signaling. The up-regulation of *Myh4* in DLK1-treated myotubes was consistent with analyses of hypertrophied muscle from callipyge sheep indicating *DLK1* signaling have an effect on fast-twitch myofiber formation. *DNTTIP1* positively regulated *Myh4* and negatively influenced *Myh7* luciferase activity, implying a direct effect of the transcription factor on muscle fiber switch in callipyge muscles (Fig. 9b).

*Dnttip1* is a transcriptional cofactor that negatively regulates the activity of terminal deoxynucleotidyltransferase (TdT), which is a DNA polymerase synthesizing the N-region of B and T-cell receptor genes, independent of a DNA template [59]. *Dnttip1* is ubiquitously expressed, exclusively localized in the nucleus and it encodes a protein with a helix-turn-helix and AT-hook-like motif that preferentially binds to AT-rich regions of double-stranded DNA [59, 60]. This information suggests broader roles of this protein in other tissues. Notably, the promoter region of *Myh4* gene contains two AT-rich motifs [61] which indicate that *Dnttip1* may bind to the *Myh4* promoter region to active its transcription. Moreover, DNTTIP1 is reported to interact with HDAC1 and HDAC2 during M phase [62]. Further protein structural analysis confirmed that DNTTIP1 forms a stoichiometric compex with HDAC1 and the ELM2-SANT domain and is required for the stable assembly of the cyclin A associated MiDAC complex [63]. The HDACs usually down-regulate transcriptional activity by deacetylating histones [64]. The interaction with HDACs may facilitate *Dnttip1* regulation of gene expression in muscles. The function of *Dnttip1* in muscle growth is unclear.

Both *PDE4D* and *SLC22A3* have increased expression only in hypertrophied muscles, which implied they may play roles in *DLK1*-induced muscle hypertrophy. The luciferase analysis was not performed for *Pde4d* in this study due to the confounding expression of numerous alternatively spliced transcripts that dictate subcellular localization and the specific cAMP pools that are affected [65]. *Pde4d* is a member of phosphodiesterases (PDEs), which catalyze the hydrolysis of cyclic nucleotides cAMP into the inactive substrate 5’-AMP. cAMP signaling is important for muscle hypertrophy and metabolism [66]. The β2-adrenergic receptors (β2-ARs) as cAMP inducers in skeletal muscle are of particular importance since they are major targets are β-agonists. The phenotype observed in callipyge hypertrophy is very similar to muscle growth induced by β-agonists, which includes increased muscle mass and decreased adiposity [67, 68]. Activation of *Pde4d* has been shown to be an important feedback regulation system contributing to the transient nature of the β2-ARs response and it represents a major adaptive mechanism required for physiological β2-ARs signaling [69]. Callipyge lambs do not respond to β-agonist supplementation with additional muscle hypertrophy [70]. Therefore, the up-regulation of *PDE4D* in hypertrophied muscles may be associated with a stronger response to physiological levels of adrenaline in young growing lambs.

*Slc22a3 / Oct3* (Solute carrier 22A3 / Organic cation transporter 3) is an organic cation transporter and is expressed in a wide range of tissues [71, 72]. By contrast, other two *Slc22a* gene family members, including *Slc22a1* and *Slc22a2,* are highly expressed in liver and kidney respectively [71]. *Slc22a3* knockout mice did not show overt phenotypic abnormalities indicating the loss of *Slc22a3* could be potentially compensate by other two members since they share functional similarities by transporting catecholamines and neurotoxin MPP+ (1-methyl-4-phenylpyridinium) [72–76]. Since the knockout mouse model failed to show any skeletal muscle abnormality, we suspect that *SLC22A3* is not a direct transcriptional response to *DLK1* signaling in hypertrophied muscles. Herein, it was excluded from the luciferase study. *Slc22a3* mediates the uptake of many important endogenous amines, particularly catecholamines such as norepinephrine and dopamine, and exogenous drugs, such as metformin in a variety of tissues [77–80]. Studies have shown that in excretory organs, such as kidney and liver, *Slc22a3* facilitated the uptake of organic cations across the basalateral membrane into the cell [81, 82]. *Slc22a3* is expressed in skeletal muscle [83] but its up-regulation in hypertrophied muscles is now apparent from the current investigation and our earlier studies [24, 53]. It is assumed that the high-levels of *SLC22A3* may enhance the up-take of amines including amino acids and biogenic amines that stimulate muscle growth in callipyge lambs. Further studies will be needed to explore its function in muscle development and hypertrophy.

Since *Park7* was not up-regulated in DLK1-treated cells, it may not act as a direct transcriptional response to *DLK1* signaling but may be a fundamental tertiary response to increased muscle growth. Interestingly, double muscled cattle have elevated levels of *PARK7* gene expression and had an increased proportion of white fast-twitch glycolytic fibers as well [84]. With increased *PARK7* expression in hypertrophied muscles and its regulation of *Myh4* and *Myh7* luciferase activity, it may have a physiological role in response to the DLK1-induced muscle hypertrophy. *Park7* encodes a ubiquitously expressed, highly conserved protein that was originally identified as an oncogene that transforms NIH3T3 cells in cooperation with the activated *ras* gene [85]. *Park7* has been associated with diverse biological processes including oxidative stress response, transcriptional regulation and cell survival [86–88]. Earlier in vitro study showed the significantly larger diameters and more total sarcomeric myosin expression in the *Park7* (+/+) myotubes than in the *Park7* (−/−) myotubes partially due to the altered activity of the PI3K/AKT pathway [27]. The up-regulation of *PARK7* in callipyge lambs may lead to the enhanced activity and/or prolonged sustained activity of the PI3K/AKT pathway and in turn to increase the response of the downstream elements in the PI3K/AKT pathway to increase protein synthesis and muscle mass. Moreover, over-expression of constitutively active AKT resulted in the hypertrophy of glycolytic myofibers not oxidative myobfiers [89]. This study was supported by the identification a regulatory cascade that regulated AKT activation to drive the metabolic and contractile specification of fast-twitch muscle fibers [90]. Therefore, *Park7* induced increase in *Myh4* luciferase activity could be a result of enhanced activation of AKT.

*METTL21E* up-regulated *Myh7* and down-regulated *Myh4* luciferase activity. Although this pattern is different from *DLK1*, *METTL21E* may still have a physiological role in callipyge muscle hypertrophy since it is consistently up-regulated in hypertrophied muscles in a manner similar to *DLK1*. *Mettl21e* has nuclear localization, but it mostly accumulated in perinuclear cytoplasm (Additional file 8). Hence, it may not act as a direct transcriptional response to *DLK1* signaling. The results from the luciferase assay suggested *METTL21E* may not regulate the myosin heavy chain gene expression, but it may be involved in other biological activities. Accordingly, skeletal muscle has the highest expression level of *Mettl21e* than any other tissues in mouse (http://biogps.org/#goto=genereport&id=403183). *Mettl21e* encodes a methyltransferase domain similar to members of the S-adenosylmethionine (SAM) -dependent methyltransferase family [91]. This family of methyltransferases catalyzes the transfer of a methyl group (CH3) from a donor, generally S-adenosyl-L-methionine (AdoMet), to various acceptor molecules [92, 93]. In human, there is no *METTL21E* ortholog, but the bovine *METTL21E* gene shares 51% similarity to human *METTL21C*. Due to the species specificity, the function of *Mettl21e* is not well defined. However, thousands of substrates of SAM-dependent methyltransferases has been identified [94]. These substrates include nucleic acids for regulation of gene expression, DNA or proteins for repair or control of signal transduction pathways, hormones and neurotransmitters, and biosynthetic intermediates to produce secondary metabolites [93]. The bioactivities of the substrates may indicate diverse functional roles of *METTL21E* during the callipyge muscle development.

## Conclusions

In summary, the present study extended knowledge on the genes involved in muscle hypertrophy in the callipyge lambs. The study provided additional support that *RTL1* alone was insufficient to induce muscle hypertrophy and concluded that *DLK1* was the likely primary inducer of the hypertrophy phenotype. From analyses of *DNTTIP1, PARK7, SLC22A3, PDE4D* and *METTL21E* expression, it is proposed that *DNTTIP1* and *PDE4D* are the secondary effector genes responding to *DLK1* signaling (Fig. 9b) and *DNTTIP1* may respond DLK1 signaling to modulate myosin heavy chain gene expression. We also discovered *PARK7* can play a role in muscle fiber switching. Identification of the genes and the signaling pathway that cause the callipyge phenotype will enrich the understanding of postnatal muscle growth in sheep and potentially has application to other livestock species used for meat production.

## Additional files

Additional file 1: Quantitative PCR primer sequences and amplification conditions. (XLSX 13 kb)

Additional file 2: The plasmids compositions of effector luciferase assay. (XLSX 9 kb)

Additional file 3: Quantitative analysis of gene expression in 7 muscles (log). (XLSX 16 kb)

Additional file 4: Analysis of variance of genotype effects in qPCR analysis. (XLSX 14 kb)

Additional file 5: Transcript abundance of *RPLP0* in 7 muscles. Least square means and standard errors for log transcript abundance are shown for each muscle and genotype, callipyge (+/C) and normal (+/+). The hypertrophied muscles are LD, SM, ST, and TB and the non-hypertrophied muscles are SS, IS and HT. *RPLP0* was used as a control to confirm equivalent RNA input into cDNA synthesis and qPCR assays. No significant differences were detected between the two genotypes in all the muscles analyzed. (PDF 21 kb)

Additional file 6: Cellular localization of DNTTIP1. C2C12 cells were transfected with pDNTTIP1-pcDNA3.2 /V5 construct and stained with anti-DNTTIP1 antibody (red), anti-V5 epitope tag antibody (green) and DAPI (nuclei, blue). The merged cells (orange) showed the detection of the same cells by anti-DNTTIP1 and anti-V5 antibodies. DNTTIP1 localized exclusively in nucleus in C2C12 cells. (PDF 43 kb)

Additional file 7: Cellular localization of PARK7. C2C12 cells were transfected with pPARK7-pcDNA3.2 /V5 construct and stained with anti-PARK7 (green), anti-V5 epitope tag (red) and DAPI (nuclei, blue). The merged cells (orange) showed the detection of the same cells by anti-PARK7 and anti-V5 antibodies. PARK7 localized both in the cytoplasm and nucleus in C2C12 cells. (PDF 69 kb)

Additional file 8: Cellular localization of METTL21E and GW-CAT. C2C12 cells were transfected with pMETTL21E-pcDNA3.2 /V5 construct (A to C) or pGWCAT- pcDNA3.2/V5 (D to F) and stained with anti-METTL21E or anti-GW-CAT antibody (green), and DAPI (nuclei, blue). METTL21E (A to C) localized both in the cytoplasm and nucleus but mostly accumulated in cytoplasm in C2C12 cells. GW-CAT (D to F) localized mostly in nucleus. (PDF 51 kb)

## Abbreviations

ANOVA: Analysis of variance
DIO3: Deiodinase, Iodothyronine, type III
DLK1: Delta-like homolog 1
DNTTIP1: Deoxynucleotidyl Transferase Interacting Protein 1
HT: Heart
IS: Infraspinatus
LD: longissimus dorsi
LPL: Lipoprotein Lipase
MEG3: Maternal Expressed Gene 3
MEG8: Maternal Expressed Gene 8
METTL21E: Protein-Lysine Methyltransferase 21E
MYH1: Myosin Heavy Chain 1
MYH2: Myosin Heavy Chain 2
MYH4: Myosin Heavy Chain 4
MYH7: Myosin Heavy Chain 7
PARK7: Parkinson Protein 7
PDE4D: cAMP specific Phophodiesterase 4D
PFKM: Phosphofructokinase, muscle
RPLP0: Ribosomal Protein, large P0
RTL1: Retrotransposon-like 1
RTL1AS: RTL1 antisense
SLC22A3: Solute Carrier Family 22 Member 3
SM: Semimembranosus
SS: Supraspinatus
ST: Semitendinosus
TB: Triceps brachii
