# Supplementary figures and images for: Identification of genes directly responding to DLK1 signaling in Callipyge sheep
Source: BMC Genomics. 2018 Apr 24;19:283. doi: 10.1186/s12864-018-4682-1 (PMC5937834; doi:10.1186/s12864-018-4682-1)

Log Transcript Abundance

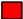 +/C  
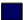 +/+

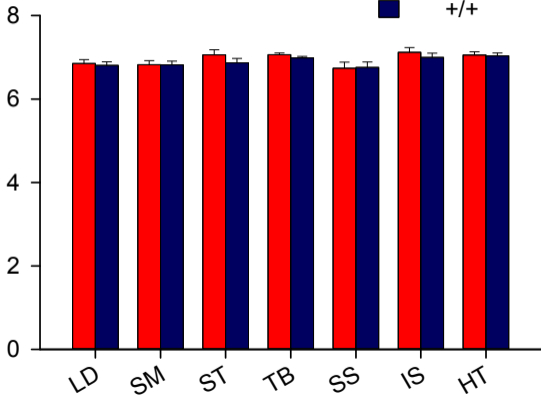

Supplement: Supplementary file 5 — Transcript abundance of RPLP0 in 7 muscles. Least square means and standard errors for log transcript abundance are shown for each muscle and genotype, callipyge (+/C) and normal (+/+). The hypertrophied muscles are LD, SM, ST, and TB and the non-hypertrophied muscles are SS, IS and HT. RPLP0 was used as a control to confirm equivalent RNA input into cDNA synthesis and qPCR assays. No significant differences were detected between the two genotypes in all the muscles analyzed. (PDF 21 kb) [file 12864_2018_4682_MOESM5_ESM.pdf]

V5 epitope tag / DNTTIP1 / DAPI

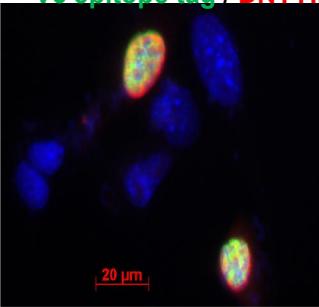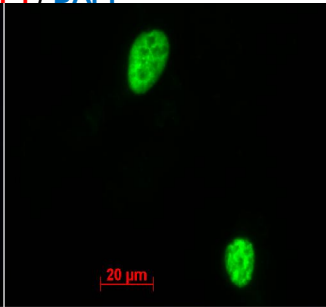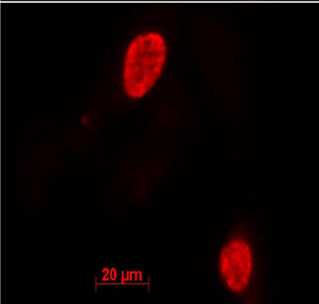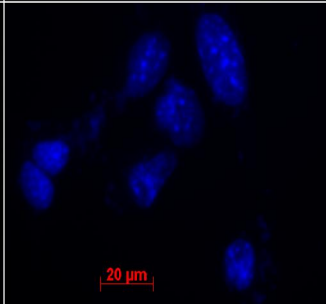

Supplement: Supplementary file 6 — Cellular localization of DNTTIP1. C2C12 cells were transfected with pDNTTIP1-pcDNA3.2 /V5 construct and stained with anti-DNTTIP1 antibody (red), anti-V5 epitope tag antibody (green) and DAPI (nuclei, blue). The merged cells (orange) showed the detection of the same cells by anti-DNTTIP1 and anti-V5 antibodies. DNTTIP1 localized exclusively in nucleus in C2C12 cells. (PDF 43 kb) [file 12864_2018_4682_MOESM6_ESM.pdf]

PARK7 / V5 epitope tag / DAPI

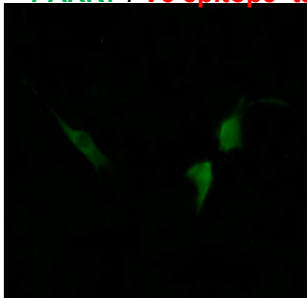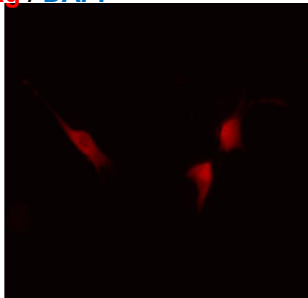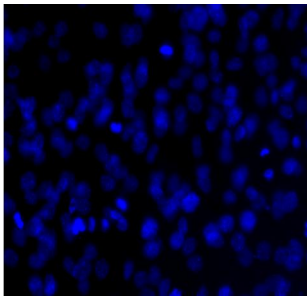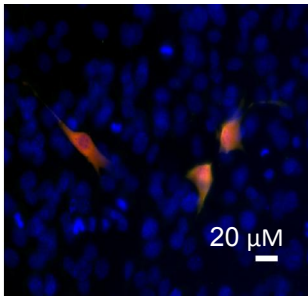

Supplement: Supplementary file 7 — Cellular localization of PARK7. C2C12 cells were transfected with pPARK7-pcDNA3.2 /V5 construct and stained with anti-PARK7 (green), anti-V5 epitope tag (red) and DAPI (nuclei, blue). The merged cells (orange) showed the detection of the same cells by anti-PARK7 and anti-V5 antibodies. PARK7 localized both in the cytoplasm and nucleus in C2C12 cells. (PDF 69 kb) [file 12864_2018_4682_MOESM7_ESM.pdf]

# METTL21E or GW-CAT / DAPI

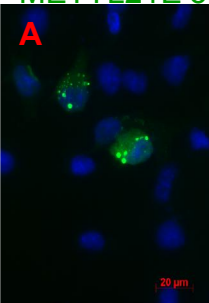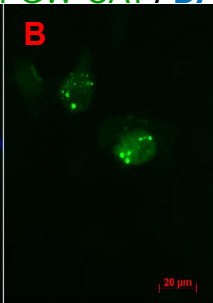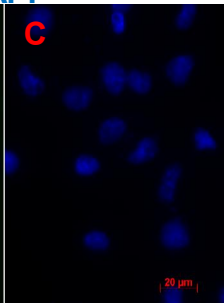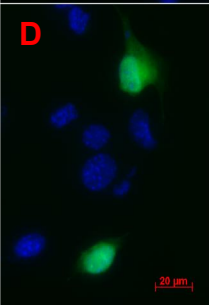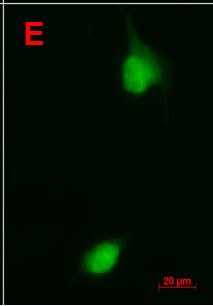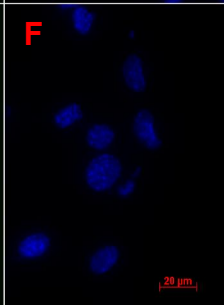

Supplement: Supplementary file 8 — Cellular localization of METTL21E and GW-CAT. C2C12 cells were transfected with pMETTL21E-pcDNA3.2 /V5 construct (A to C) or pGWCAT- pcDNA3.2/V5 (D to F) and stained with anti-METTL21E or anti-GW-CAT antibody (green), and DAPI (nuclei, blue). METTL21E (A to C) localized both in the cytoplasm and nucleus but mostly accumulated in cytoplasm in C2C12 cells. GW-CAT (D to F) localized mostly in nucleus. (PDF 51 kb) [file 12864_2018_4682_MOESM8_ESM.pdf]
